# Treatment Approach for Maxillary Hypoplasia in Cleft Patients: Class III Elastics with Skeletal Anchorage (Report of Two Cases)

**Published:** 2016-07

**Authors:** Arezoo Jahanbin, Mozhgan Kazemian, Iman Saeedi-Pouya, Neda Eslami, Hooman Shafaee

**Affiliations:** 1 *Dental Research Center, School of Dentistry, Mashhad University of Medical Sciences, Mashhad, Iran*; 2 *Oraland Maxillary Diseases Research Center, School of Dentistry, Mashhad University of Medical Sciences, Mashhad, Iran.*; 3 *Department of Orthodontics, Mashhad University of Medical Sciences, Mashhad, Iran.*; 4 *Oral and Maxillofacial Diseases Research Center, School of Dentistry, Mashhad University of Medical Sciences, Mashhad, Iran.*

**Keywords:** Cleft lip and palate, Skeletal anchorage, Maxillary advancement

## Abstract

**Introduction:**

Treatment of cleft lip and palate patients requires a multidisciplinary plan. These patients usually have a hypoplastic maxilla due to the prior surgical scars. Orthognathic surgery to advance the maxilla in these patients is not very efficient; therefore, orthopedic interventions during an appropriate age seems to be essential.

**Case Report:**

In this article, two cleft lip and palate patients have been treated with Class III elastics anchored to the maxillary posterior and mandibular anterior miniplates in order to induce maxillary advancement.

**Conclusion:**

Both cases showed a significant improvement in their profiles with minimal dentoalveolar compensations. A counterclockwise rotation of the mandible occurred.

## Introduction

Cleft lip and palate is one of the most prevalent congenital deformities. Its incidence has been reported to be 1:700 in European countries and in the USA (1). This deformity is multifactorial and in most cases, no etiologic factor has been discovered (1).

Children with unilateral or bilateral cleft lip and palate are usually at risk for poor facial growth. They are prone to developing midfacial retrusion related to maxillary hypoplasia or growth retardation secondary to excessive palatal scarring. Usually, this results in 3-Dimentional deficiencies and an anterior dental crossbite or severely rotated maxillary incisors which may conclude to a tip to tip relationship with the mandibular incisors. Depending on the age of the patient and the extent of their midfacial development, some of these early problems can be corrected using midfacial or orthopedic protraction forces. These forces increase growth at the circummaxillary sutures as the maxilla is repositioned anteriorly (2).

Young patients with maxillary hypoplasia are usually treated with a facemask. A heavy force is applied on the maxilla to stimulate its growth in a forward and downward direction and to redirect mandibular growth (3-7). However, facemask therapy results in a posterior rotation of the mandible and increased vertical dimension of the force. (3,4,8) Moreover, as the forces were applied on the teeth, dental compensations were observed (3,9). It has been claimed that using facemask in conjunction with skeletal anchorage reduced the aforementioned complications and enhanced its skeletal efficacy (10-12). Beak et al. reported that facemask attached to the skeletal anchorage can be an effective alternative treatment modality for maxillary hypoplasia with minimal unwanted side effects in cleft patients (13).

It should be noted that wearing the facemask is usually limited to 14 hours per day at best; but,class III elastics attached to titanium miniplates as an anchorage device, offer the possibility to apply full-time orthopedic forces between the maxilla and mandible, while reducing dentoalveolar compensations(14). To the best of our knowledge, this treatment procedure was not attempted in cleft lip and palate patients. Therefore, in our presented cases, two miniplates were inserted in the anterior part of the mandible in the canine areas and two miniplates were inserted in the posterior part of the maxilla. Class III elastics were used between them in order to correct maxillary deficiency.

## Case Reports

*Case 1:*

**Fig 1:**
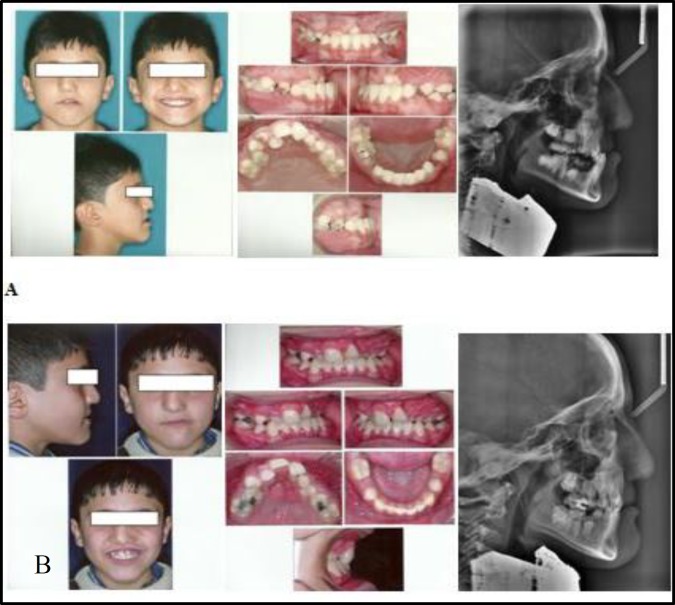
Patient1. A, Pre-treatment facial and intraoral photographs. B, Post-treatment facial and intraoral photographs

A nine-year-old boy with unilateral cleft lip and palate was referred to the Department of Orthodontics of Mashhad University of Medical Sciences (Iran). At the beginning of the treatment, study models, panoramic radiograph and lateral cephalogram as well as facial and intraoral photographs were taken. The patient presented with a concave facial profile, anterior cross bite (overjet: -4mm), and bilateral posterior cross bite. Cephalometric analysis showed skeletal Class III malocclusion with maxillary hypoplasia and steep mandibular plane angle (Fig.1). He had no medical problems.

*Treatment Procedures:*

Initially, a W-arch expander constructed from a 0.9 mm stainless steel wire was cemented on the molars. This device was activated 3 mm/month to overcorrect the posterior crossbite. Next, two L-shape miniplates (General implants, GmbH Deutschland, Germany) were placed at the zygomatic buttress under local anesthesia by a maxillofacial surgeon and fixed with three miniscrews, the distal ends of the miniplates were exposed through the attached gingiva between the upper first permanent molars and second premolars to control vector of elastic traction. In the mandible, two L-shape miniplates (General implants, GmbH Deutschland, Germany) were placed under local anesthesia and were fixed by three miniscrews. The terminal ends of the miniplates were exposed between the lower central and lateral incisors. The ideal position for the insertion of the miniplates was evaluated using a panoramic radiograph in order to avoid damage to the roots of the adjacent teeth and mental foramen.

Four weeks after the placement of the miniplates, their mobility was checked by the surgeon and orthodontic latex elastics (3/16′′ heavy size on both sides-American Orthodontics, Sheboygan, USA) were attached to the hooks of the miniplates to generate approximately 100 g of force on each side. 

**Fig 2:**
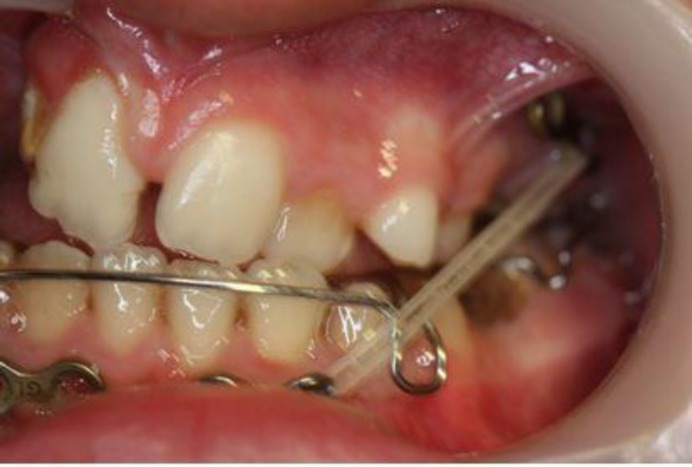
The application of intermaxillary elastic to the miniplates

The patient wore a tightly fitting and well-retained lower removable appliance in order to disclude the upper and lower jaws during maxillary traction. The patient was instructed to wear the removable appliance full-time except for eating, contact sports, and tooth brushing; he was also told to replace the elastics every day (Fig.2). The traction force was doubled after 1 month, and the final 250g of force per side was reached after 2 months. Skeletal and soft tissue analyses were performed on pre-treatment and post-treatment cephalograms (15,16).

*Treatment Results:*

After four month of active treatment, a positive overjet and a significant improvement in the patient’s profile were achieved (Fig.1). Post-treatment cephalo- metric tracing of patient 1, showed a favorable increase of 4° and +4.5 mm in ANB and wits appraisal, respectively. (Table.1). Also, soft tissue analysis of the patient revealed a favorable forward movement of the upper lip (Table.2). Minimal movements of the upper and lower incisors were observed (Table.3). Pre- and post-treatment cephalometric superimpositions on the anterior cranial base are showed in Figure 4.

**Table 1:** Skeletal Cephalometric analysis

**Skeletal ** **measurements**	**Patient 1**		**Patient 2**	
	**Pre-treatment**	**Post-treatment**	**Pre-treatment**	**Post-treatment**
SNA (˚)	69	73	73	76
SNB (˚)	73	73	75	75
ANB (˚)	-4	0	-2	+1
Wits (mm)	-5	-0.5	-3	0
SN-Platal plane (˚)	4.5	3.5	5	4.5
SN-Occlusal Plane (˚)	20	18	24	15
Go.Gn-Sn	42	40	44	39
FMA (˚)	34	31	35	30

**Table 2:** Soft-tissue analaysis

**Soft-tissue measurements**	**Patient 1**		**Patient 2**	
	**Pe-treatment**	**Post-treatment**	**Pre-treatment**	**Post-treatment**
Nasolabial angle (˚)	105	90	120	110
Upper lip prominence (mm)	0	2	0	1
Interlabial gap(mm)	1	1	4	1
Angle of facial concavity (˚)	-6	-1	-5	-1
H – line angle	3	7	4	7
Upper sulcus depth (mm)	3	4	4	5
Nasolabial angle	105	90	120	110

**Table 3:** Dental analysis

**Dental measurements**	**Patient 1**		**Patient 2**	
	**Pe-treatment**	**Post-treatment**	**Pre-treatment**	**Post-treatment**
Over jet (mm)	-4	0	-5	0
Overbite	2	2	-7	0
U1 to SN	79	79	82	85
IMPA	84	84	86	85

*Case 2:*

The second case was an eleven-year-old boy with bilateral cleft lip and palate. Similar to case 1 clinical and radiographic examinations were performed. The patient presented with concave facial profile, anterior cross bite (overjet: -5 mm), anterior open bite (overbite:-7 mm), and posterior crossbite. This case also showed skeletal Class III malocclusion due to maxillary hypoplasia and steep mandibular plane angle (Fig. 2, Table.1).

*Treatment Procedures:*

All the treatment steps were similar to the case 1.

*Treatment Results:*

**Fig3:**
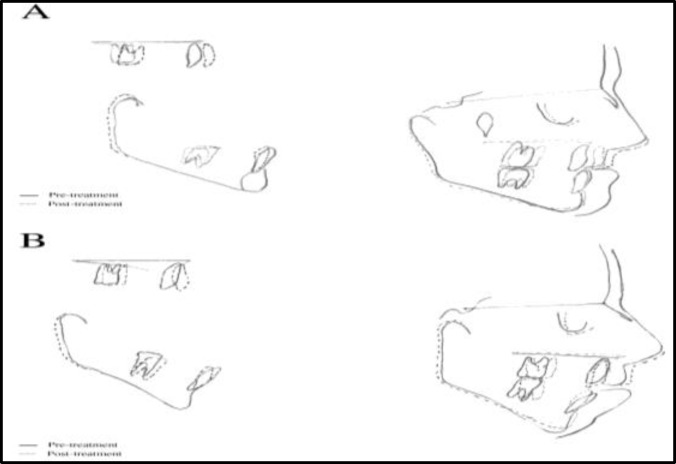
Superimposition of lateral cephalograms.  A: Case 1 B: Case 2

**Fig4:**
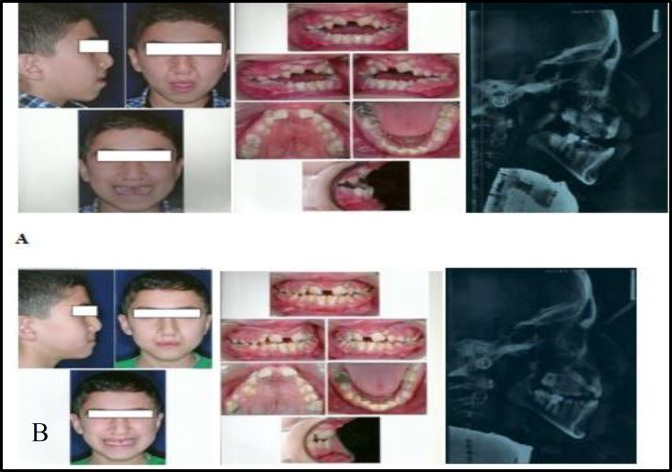
Patient2. A, Pre-treatment facial and intraoral photographs. B, Post-treatment facial and intraoral photographs.

After four month of active treatment a significant improvement in the patient’s profile was achieved. The anterior open bite was also reduced significantly (Fig.4). The over jet of the patient was increased from -5 to 0. In this case, the ANB angle and wits appraisal favorably increased by 3° and +3 mm, respectively (Table.1). Also, soft tissue analysis of the patient revealed a favorable forward movement of the upper lip(Table.2).Minimal movements of the upper and lower incisors were observed (Table.3). Pre- and post-treatment cephalometric superimpositions on the anterior cranial base were demonstrated in Figure 3.

## Discussion

Before introducing TADs, orthodontists have tried growth modifications by applying orthopedic forces to the teeth (3-5). Therefore, dentoalveolar compensations rather than alterations of the facial growth were mainly responsible for improvement of malocclusion (3,9). To avoid dental compensations, titanium miniplates can be used to apply the orthopedic forces. Ahn et al. showed that the midface can be pulled forward over a mean distance of 8 mm using bone-borne traction hooks in combination with an extraoral face bow (17). Lee et al. reported that facemask therapy in conjunction with miniplates (FM/MP) induced a greater advancement of the maxilla (12), less posterior repositioning and opening rotation of the mandible, and less proclination of maxillary incisors compared to routine facemask therapy associated with rapid maxillary expansion. Kayaa et al. also reported that facemask with miniplates offer an advantage for correcting mild/moderate maxillary retrusion in class III patients (18). Beak et al. used this technique (FM/MP) to treat maxillary hypoplasia in cleft patients and they concluded that FM/MP can be an effective alternative treatment modality for maxillary hypoplasia with minimal unwanted side effects in cleft patients. Although (13), the aforementioned studies showed promising results, they still relied on facemask wear, and thus patient compliance. In this present study, we used intermaxillary elastics applied to miniplates to protract the maxilla in our cleft patients. Heyman et al. also reported that maxillary protraction with intermaxillary elastics applied to miniplates resulted in minimal dentoalveolar compensations (14). 

The elastic forces used in this method were lower than facemask therapy forces (3-5). This moderate continuous traction may be more favorable than heavy intermittent forces. In our presented cases, a significant displacement of the maxilla associated with minimal mandibular growth resulted in a clear reduction in facial concavity. Dental analysis measurements (U1-SN and IMPA) showed minimal dentoalveolar compensations. The palatal plane rotated counterclockwise moderately (Fig.3). This finding was the result of the direction of force application between the maxillary and mandibular miniplates, which were located below the center of resistance of the maxilla. Go. Gn-SN and FMA angles were decreased after treatment. In patient 2, a significant improvement in open bite has been observed. The vector of force was located superiorly to the center of resistance of the mandible and the counterclockwise rotation of the mandible may be the reason of this improvement. Soft tissue analysis showed improvement in both cases. Nasolabial angles were decreased which was as a result of maxillary protraction. Angle of facial concavity values showed an improvement in the facial soft tissue profile of both cases. The prominence of the upper lip was slightly improved in both cases.

## Conclusion

Intermaxillary elastics applied to miniplates are a promising technique for maxillary protraction with minimal dentoalveolar compensation in cleft lip and palate patients.
